# Comparison of transcriptional expression patterns of phenols and carotenoids in ‘Kyoho’ grapes under a two-crop-a-year cultivation system

**DOI:** 10.1371/journal.pone.0210322

**Published:** 2019-01-10

**Authors:** Guo Cheng, Sihong Zhou, Jin Zhang, Xiaoyun Huang, Xianjin Bai, Taili Xie, Rongrong Guo, Jinbiao Liu, Huan Yu, Linjun Xie

**Affiliations:** 1 Grape and Wine Research Institute, Guangxi Academy of Agricultural Sciences, Nanning, Guangxi, China; 2 Guangxi Crop Genetic Improvement and Biotechnology Laboratory, Nanning, Guangxi, China; Mediterranean Agronomic Institute of Chania, GREECE

## Abstract

To fully utilize the characteristic climatic conditions in the southern region of China, a two-crop-a-year cultivation system technique for ‘Kyoho’ grape was developed during the past decade. After summer harvest in June, appropriate pruning and chemical treatments promote flowering and fruiting, which enables a second harvest in late December. Due to climatic differences between the two crop growing seasons, grape phenol and carotenoid metabolism differ greatly. The reported study analyzed the transcriptome of the carotenoid and phenylpropanoid/flavonoid pathways in grapes at four different stages during the two growing seasons. Compared with those in summer grapes, expression levels of the majority of genes involved in the carotenoid metabolic pathway in winter grapes were generally upregulated. This result was associated with lower rainfall and much more abundant sunlight during the second growing season. On the other hand, summer cropping strongly triggered the expression of upstream genes in the phenylpropanoid/flavonoid pathway at E-L 33 and E-L 35. Transcript levels of flavonoid 3’,5’-hydroxylase (F3’5’H), flavonoid 3’-hydroxylase (F3’H), flavonoid 3-hydroxylase (F3H) and glutathione S-transferase (GST) were upregulated in winter grapes at the mature stage. Together, these results might indicate that more flavonoids would be synthesized in ripe winter grapes during the mature stage of the second crop under much drier conditions, longer sunlight hours and lower temperature. These data provide a theoretical foundation for the secondary metabolism of berries grown under two-crop-a-year cultivation systems.

## Introduction

The regions of southern China are considered to be suboptimal for grape cultivation because of extremely high temperature and concentrated rainfall [1]. Fortunately, because of the promotion of rain shelter cultivation technology and two-crop-a-year cultivation in recent years, southern China has become a booming grape production region [2]. Statistical data show that the total cultivated area of Guangxi (a province in southern China) has increased by three times, and the annual production value has increased from 246 million to 2.6 billion yuan (RMB) since two-crop-a-year techniques have applied. In most parts of Guangxi, the active accumulated temperature of the first half and second half of a year exceeds 3000°C accumulative temperature, which can meet the needs of outdoor grape growing for two seasons. In addition, the rainfall and extremely high temperature are lower during the second half of the year than during the first half of the year. Moreover, greater temperature differences between day and night during the second half of the year than during the first half of the year, and more sunlight hours occur in the former [3]. Therefore, the quality of winter grapes is much better than that of summer grapes for the same cultivar, which is mainly manifested in the greater content of anthocyanins and soluble solids concentration [4,5].

Carotenoids are a family of C40 isoprenoid pigments that have critical functions in plants, including harvesting light during photosynthesis and providing cleavage products such as the well-known phytohormone abscisic acid (ABA) [6]. The carotenoid cleavage dioxygenase (CCD) enzyme catalyzes the formation of carotene into norisoprenoids. In grape berries, most norisoprenoids are released to a free state by glycosidase or acid hydrolysis from flavorless glycosidically bound forms; these free-state norisoprenoids have extremely low perception thresholds and pleasant floral characteristics [7]. ABA generally plays important roles in regulating the onset of veraison and maturity in grapes [8]. The enzyme 9-*cis*-epoxycarotenoid dioxygenase (NCED) is thought to be the key enzyme involved in ABA biosynthesis.

Polyphenolic compounds play an important role in grape quality. Phenolic compounds containing flavonoids and nonflavonoids, anthocyanins, flavonols, and flavan-3-ols belong to flavonoids, whereas nonflavonoids include stilbenes, hydroxybenzoic and hydroxycinnamic acids. All of these compounds are derived from the phenylpropanoid/flavonoid pathway. The concentration of phenolic compounds in grapes depends on the variety and is also influenced by viticulture techniques and climatic conditions [9–11]. The influence of two growing seasons on the phenolic compound composition and concentrations in grapes has been studied, and the results showed that the concentrations of phenolic compounds in winter grapes were significantly greater than those in summer grapes [12].

The climatic conditions in southern China constitute the foundation of two-crop-a-year cultivation development. This technique exploits the rich heat and sunlight during the second half of the year. Thus, the effects of climatic conditions on grape quality are emphasized in two-crop-a-year research. Many studies have shown that both phenolic compounds and carotenoid metabolism in grape berries are greatly influenced by climatic conditions [13–14]. High temperature often increases the degradation of anthocyanins, resulting in poor coloration [15–16]. Additionally, the carotenoid metabolic flux in grapes is influenced by distinct climatic conditions among wine regions [17].

The two-crop-a-year cultivation system has solved the problems of budbreak in the spring, flower bud differentiation during the second season and fruit maturation, but poor coloration and insufficient flavor of summer grapes as well as differences in grape quality between the two crops have not been explained thoroughly. Previous studies have shown that flavonoid composition, content and metabolism are often distinct between the two crops [12,17]. However, a transcriptomic analysis of grape phenolic compounds and carotenoids in two crops grown within the same year has not been reported from a climatic difference perspective.

Thus, the present research was designed to analyze the transcriptome of phenolic compounds and carotenoids in summer and winter grapes of *Vitis labrusca* × *Vitis vinifera* L. cv. ‘Kyoho’ by RNA-seq. This paper provides new insights into the understanding of the mechanisms of secondary metabolism influenced by the growing season.

## Materials and methods

### Experimental vineyard and two-crop-a-year viticulture practices

This experiment was conducted during two growing seasons in 2016 on 4-year-old self-rooted ‘Kyoho’ grapevines in the vineyards of the Grape and Wine Research Institute, Guangxi Academy of Agricultural Sciences, located in Nanning, Guangxi Province (22°, 36’39”N, 108°, 13’51”E). The vines in this vineyard were managed on a canopy frame with a single trunk and were planted in north-south-oriented rows spaced 2.0 m (between vines) × 6.0 m (between rows). Nutrition, pest, water and fertilizer management was carried out in accordance with uniform standards for two-crop-a-year as previously described [18].

**Fig 1:**
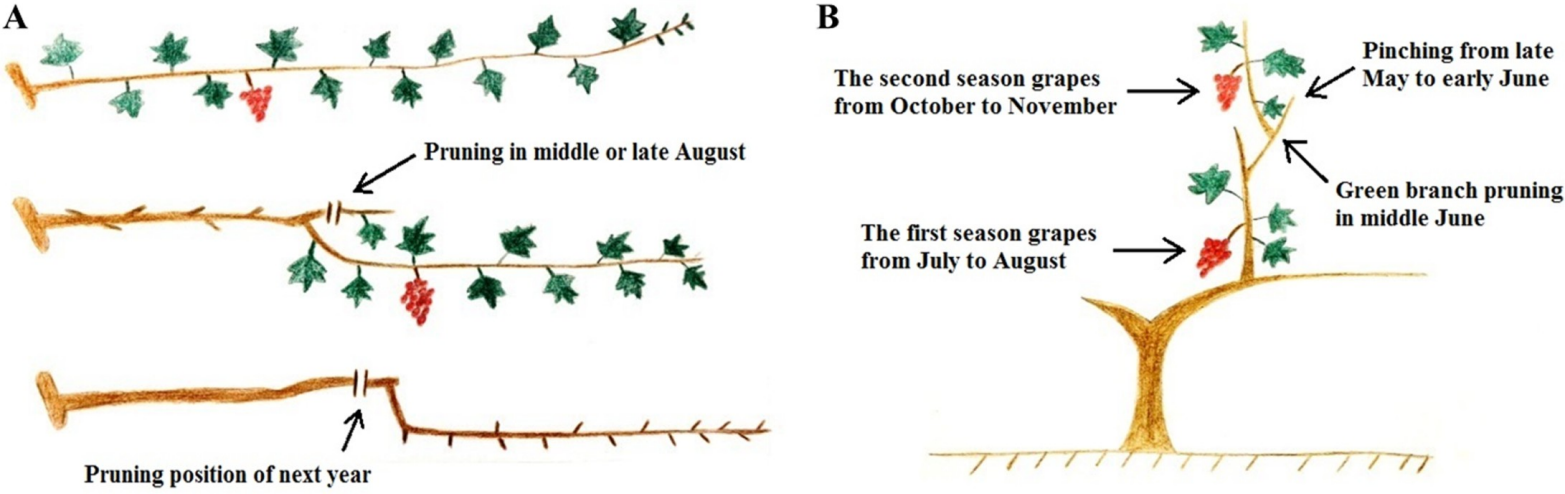
Nonoverlap (A) and overlap (B) cultivation systems.

The key techniques of two-crop-a-year cultivation systems include dormancy breaking, rain shelter cultivation, soil management, irrigation, fertilizer, pruning, flower and fruit management, and disease and pest control. Two-crop-a-year cultivation systems involve two modes referred to as nonoverlap and overlap cultivation systems (Fig 1).

In two-crop-a-year grape cultivation systems, the growing season for the first crop usually occurs from February to June, and that of the second crop occurs from August to December. The nonoverlap cultivation system is suitable for regions where the mean annual temperature > 20°C (Fig 1A), and the overlap cultivation system is suitable for regions where the mean annual temperature < 20°C (Fig 1B). In this research, the experimental vines were grown in a nonoverlap cultivation system.

### Berry sampling and physical chemical index analysis

Grape berries in three biological replicates were collected at four E-L stages [19]: berries still hard and green (E-L 33), the onset of veraison (E-L 35), berries not quite ripe (E-L 37), and the harvest stage (E-L 38). For each biological replicate, 150 berries were randomly separated from at least 100 clusters within 9 vines. The sampling time was fixed at 9:00 to 10:00 a.m., and three biological replicates were collected via the same method at each sampling date.

After being transported to the laboratory, a subsample of 100 berries from each biological replicate was subjected to physiological measurements, including berry fresh weight, total soluble solids (TSS) content and titratable acidity (TA). The remaining berries were immediately frozen in liquid nitrogen and transported to the laboratory on dry ice for transcriptional analysis. The TSS concentrations in the juices were measured with a digital pocket handheld refractometer (Digital Hand-held Pocket Refractometer PAL-1, Atago, Tokyo, Japan), and the TA was determined by titration with NaOH to the end point of pH 8.2 and was expressed as tartaric acid equivalents [16].

### Transcriptome sequencing and data analysis

A subsample of 50 berries was randomly selected from each biological replicate for RNA extraction. Transcriptome sequencing and data analysis were performed as described previously [20]. Heatmap visualizations were performed using MetaboAnalyst 3.0. From the 24-sample transcriptome analysis, we identified 175.81 Gb of clean data, and each sample produced at least 6.02 Gb. The base percentage was greater than 86.47%. The contrast efficiency of all the sample clean reads and specified reference genomes was between 58.69% and 67.16%. Based on the experimental results, alternative splicing determination, genetic structural optimization analysis, and novel gene discovery were performed. According to the expression levels in the different samples, different genes were identified, and gene functional annotation and enrichment analysis were performed. Gene expression levels were estimated via the fragments per kilobase of transcript per million fragments mapped (FPKM). The formula is as follows:

$$\mathrm{FPKM}=\frac{\mathrm{cDNA}\;\mathrm{Fragments}}{\mathrm{Mapped}\;\mathrm{Fragments}\;(\mathrm{Millions})\times \mathrm{Transcript}\;\mathrm{Length}\;(\mathrm{kb})}$$

### Validation of RNA-seq based quantitative real-time PCR

**Fig 2:**
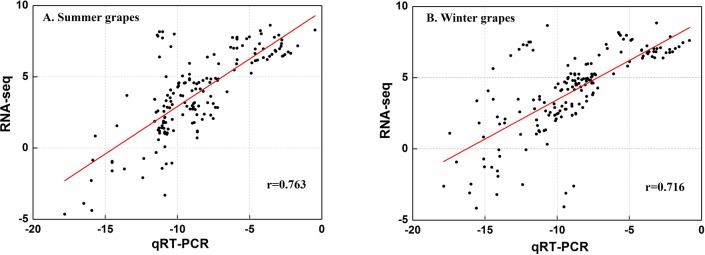
**Validation of the gene expression results of qRT-PCR and RNA-seq in the summer crop (A) and winter crop (B)**. The data were obtained from fourteen unigenes at four developmental stages. The expression values of both qRT-PCR and RNA-seq were log2 transformed. Coefficients of linear regression were also calculated.

To verify the reliability of the RNA-Seq-based transcript quantification, quantitative real-time PCR (qRT-PCR) was carried out using 14 genes related to the carotenoid and phenol pathways. The remnant RNAs from the RNA-seq experiment were used to synthesize cDNA, after which qRT-PCR was performed as previously described in [20–21]. *VvUbiquitin1* and *VvActin* were selected as two reference genes for qRT-PCR. The sequences of the specific primers used are provided in the supplementary data (S1 Table). All reactions were run in triplicate, and the normalized relative expression levels of the target genes were calculated by 2^−ΔCt^, where ΔCt = Ct (target gene) - Ct (geometric mean of two reference genes) and Ct is the mean cycle threshold [22]. Pearson correlation coefficients (p-value ≤ 0.01) were calculated to assess the correlations between the different expression patterns obtained by qRT-PCR and RNA-seq. The correlation coefficients of 0.763 for summer grapes and 0.716 for winter grapes indicated the reliability of the results of the RNA-Seq-based gene expression (Fig 2).

### Statistical analysis

Significant differences were determined when p < 0.05 according to independent t-tests. Partial least squares-discriminant analysis (PLS-DA) was performed with MetaboAnalyst 3.0, and statistical analysis was performed with SPSS (SPSS Inc., Chicago, IL, USA) for Windows, version 20.0. Line graphs were constructed with Origin 8.0 software (OriginLab Corporation, Northampton, MA, United States).

## Results

### Meteorological characteristics

**Fig 3:**
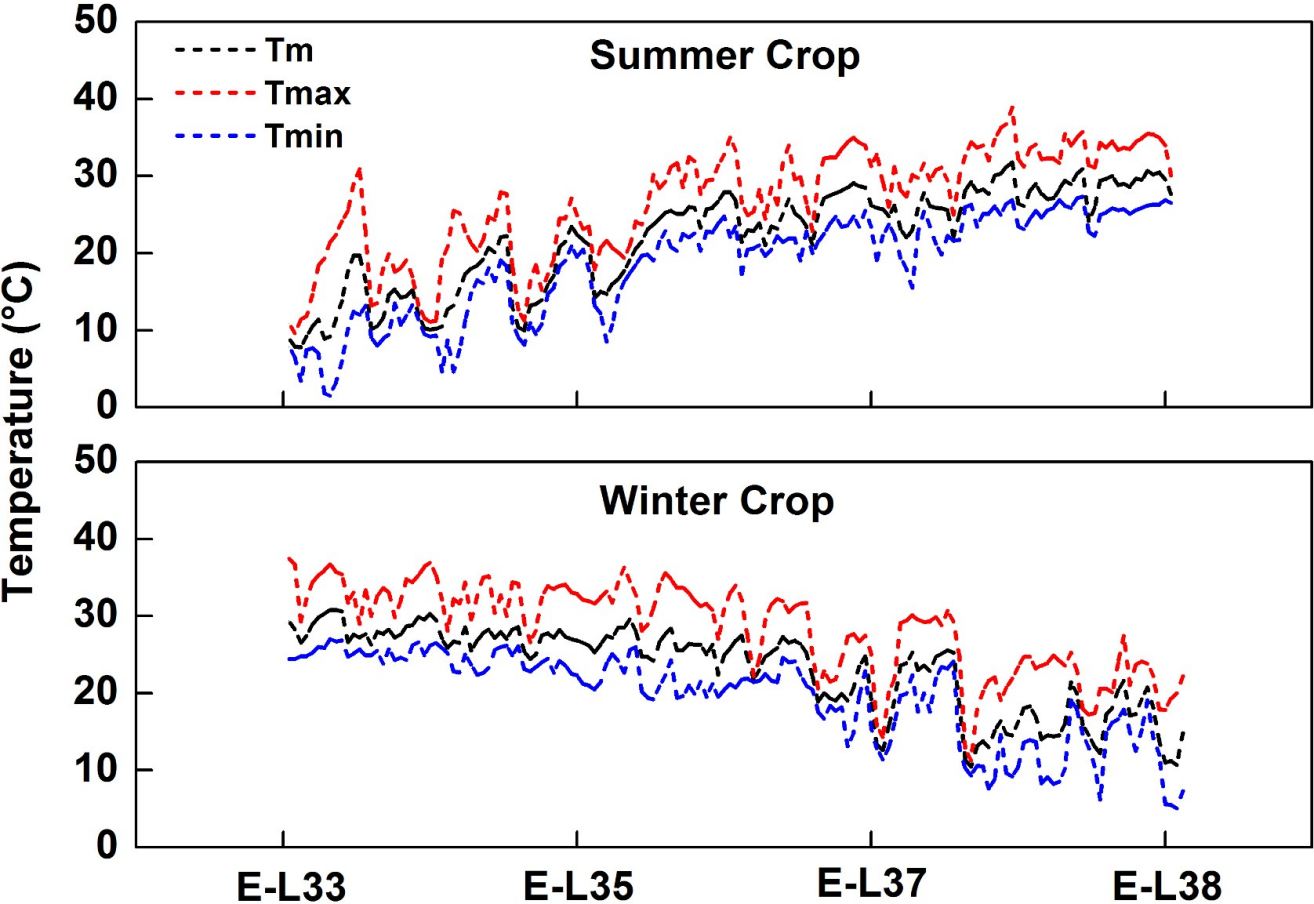
Meteorological data for both crops in 2016. Tm is the average daily temperature from February to June for the summer crop and from August to December for the winter crop. Tmax is the average daily maximum temperature from February to June for the summer crop and from August to December for the winter crop, and Tmin is the average daily minimum temperature from February to June for the summer crop and from August to December for the winter crop.

It is well known that the synthesis and accumulation of grape carotenoids and phenolic compounds are strongly affected by environmental conditions such as temperature, sunlight and rainfall [16, 23]. Table 1 displays large differences in climatic conditions between the two crop growing seasons. An active accumulated temperature of 2800°C has been suggested as the minimum for grape production [1]. In the present study, the active accumulated temperatures for both growing seasons were greater than 3200°C (Table 1), meaning that the temperatures are sufficient to guarantee normal grape maturity. In addition, the average maximum temperature from veraison to harvest during the winter season was approximately 8°C less than that during the summer season. During the growing seasons, the sunlight duration of summer grape was lower than that of winter grape. Moreover, the rainfall also differed between the two growing seasons. Specifically, there was much more rainfall during the summer growing season than during the winter growing season. For the first crop, the temperature increased gradually during the developmental stages (Fig 3). During the second growing season, the temperature decreased from E-L 33 to E-L 38. According to the berry developmental phases of the two crops, the daily maximum temperature exceeded 35°C at E-L 37 and E-L 38 for the summer crop. However, the result was the opposite for the winter crop, for which the daily maximum temperature exceeded 35°C mainly in E-L 33 and E-L 35.

**Table 1:** Climatic factors during the growing seasons of summer and winter grapes in Nanning in 2016^a^.

Year	Active-T^b^ (°C)		Sunlight-D^c^ (h)		Rain^d^ (mm)	
	Summer	Winter	Summer	Winter	Summer	Winter
2016	3274.6	3551.1	574.4	779.6	249.2	34.4
Average-30^**e**^	3242.4	3486.1	478.4	785.6	207.1	24.5

^a^ Data from the Nanning Meteorological Administration.

^b^ Active accumulated temperature during the grape growing seasons, calculated as T = ∑ti (ti ≥ 10°C); ti is the average daily temperature from February to June for the summer crop and from August to December for the winter crop.

^c^ Sunshine duration during the grape growing seasons calculated by adding the sunshine hours from February to June for the summer crop and from August to December for the winter crop.

^d^ Average monthly rainfall. Values in June for the summer crop and December for the winter crop.

^e^ Average of 30 years (from 1971 to 2000).

### Grape berry development

**Fig 4:**
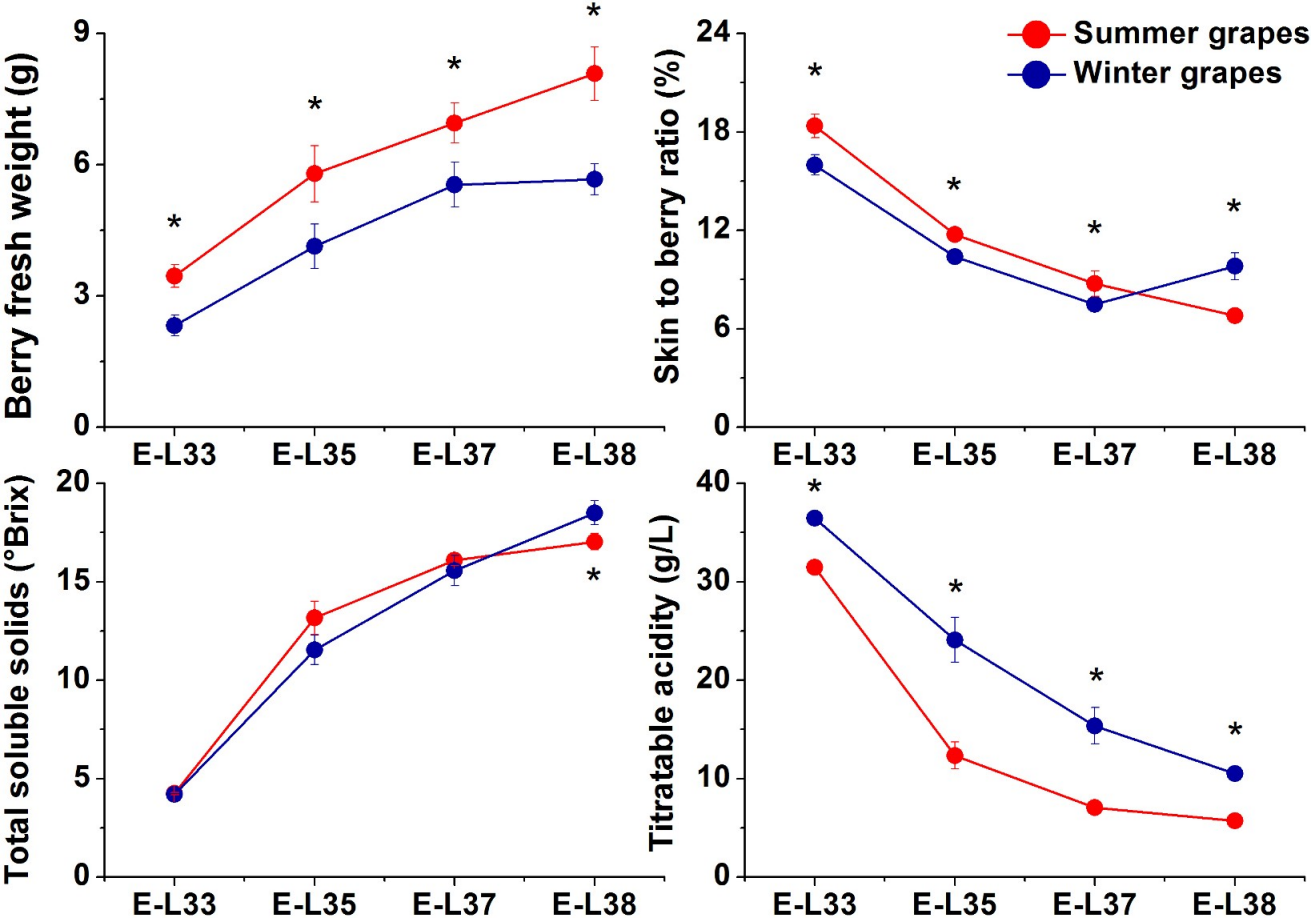
**Evolution of berry fresh weight (A), the skin-to-berry ratio (B), TSS (C) and TA (D) in grapes grown under a two-crop-a-year cultivation system in 2016**. The four points refer to stages E-L 33, 35, 37 and 38, respectively. The asterisk indicates significant differences between the samples from the same stage.

Samples that corresponded to the four stages of the E-L system [19] were measured for berry fresh weight, skin-to-berry ratio, TSS and TA (Fig 4). Summer and winter grapes presented different variation trends in physicochemical parameters. Compared with the winter grapes, the summer grapes exhibited significantly greater berry fresh weight at all developmental stages (Fig 4A). In addition, compared with the winter grapes, the summer grapes showed a significantly greater skin-to-berry ratio at E-L 33, 35 and 37 but showed the opposite result at E-L 38 (Fig 4B). As grapes ripen, their TSS content and TA gradually increases and decreases, respectively [24]. The TSS content in the winter grapes was significantly higher than that in the summer grapes at E-L 38, although no significant difference was observed at E-L 35, 36 or 37 (Fig 4C). The TA content in the winter grapes was always higher than that in the summer grapes; these contents reached approximately 11 g/L and 6 g/L titratable acids at harvest, respectively (Fig 4D).

### Global analysis of differential gene expression

**Fig 5:**
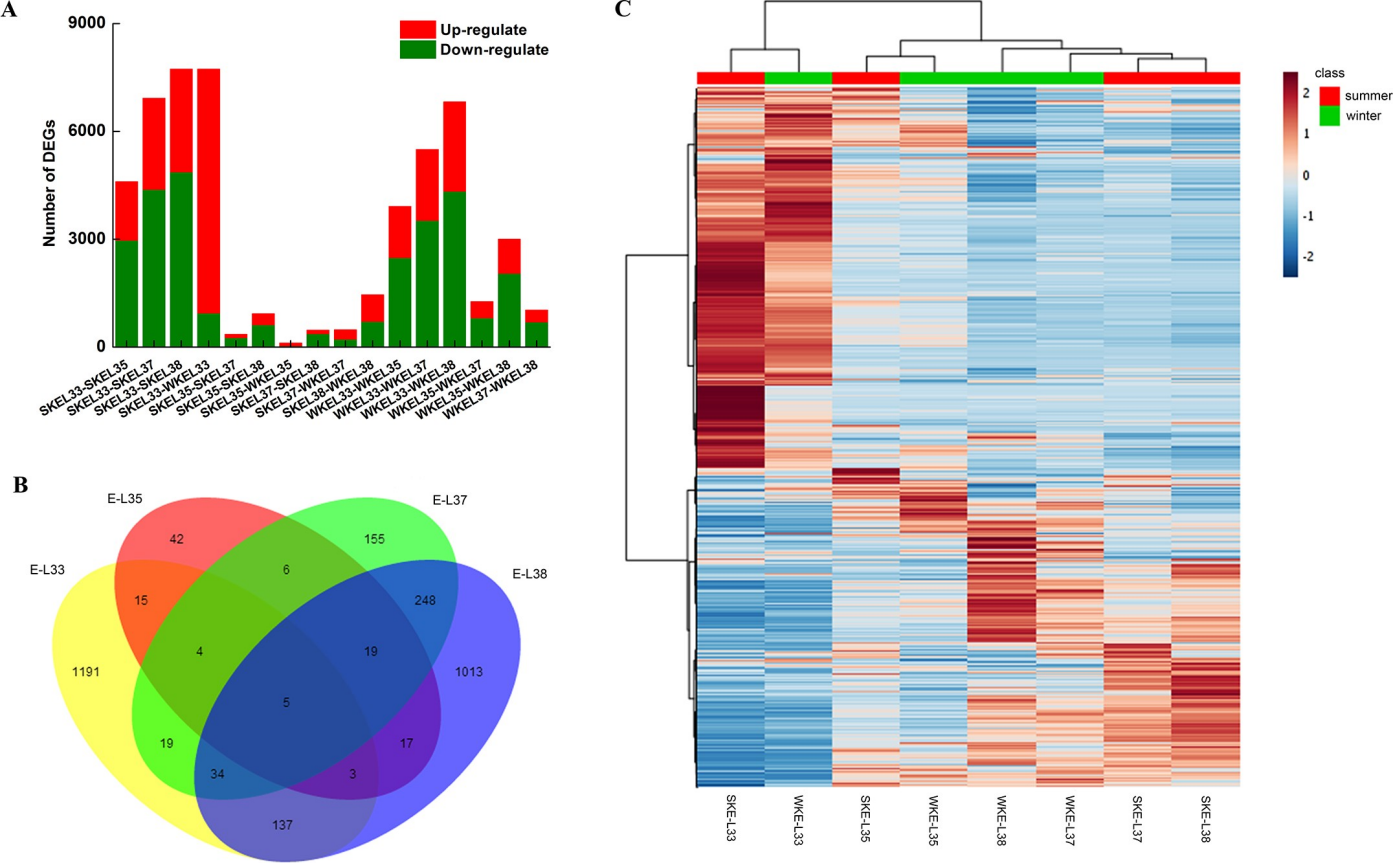
Differential gene expression in grapes under a two-crop-a-year cultivation system. (A) Numbers of DEGs in pairwise comparisons of eight samples. (B) Venn diagram showing DEG distributions. (C) Expression profile clustering.

Based on the FPKM values of the unigenes in each sample, the differentially expressed genes (DEGs) between two samples were screened (Fig 5A). Compared to the summer grapes, the winter grapes presented 6783 upregulated transcripts and 947 downregulated transcripts in the four stages. Generally, fewer transcripts were upregulated in grapes at the late stage from the same growing season than were downregulated. As shown in the Venn diagrams illustrating the relationships among DEGs at the different developmental stages (Fig 5B), 1191, 42, 155 and 1013 genes were specifically expressed in E-L 33, 35, 37 and 38, respectively, whereas only five DEGs were shared by all four stages. The DEGs from the eight samples were subsequently processed by hierarchical clustering. The results showed that samples at the same developmental stage were clustered together, indicating their similarities in gene expression (Fig 5C).

### PLS-DA

**Fig 6:**
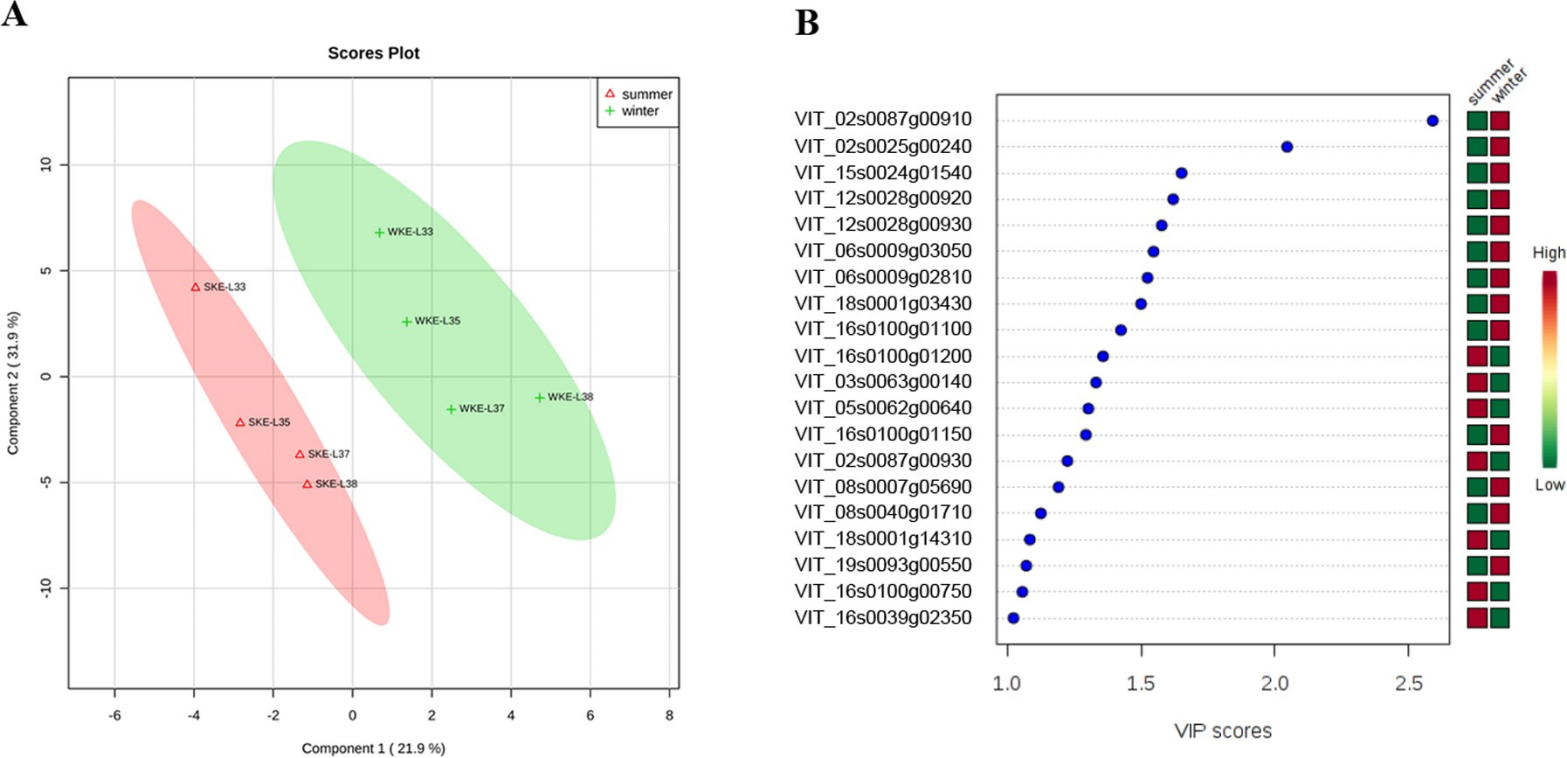
Results of PLS-DA. **(A) Score plot. (B) Selected genes based on VIP scores**. The four points refer to stages E-L 33, 35, 37 and 38, respectively.

PLS-DA was performed on the whole normalized gene expression data of the carotenoid and phenolic pathway (Fig 6). The data were normalized by the autoscaling method in MetaboAnalyst 3.0. As shown in Fig 6A, grape samples from the two seasons were completely separated from each other. Variables whose variable importance in projection (VIP) values > 1.0 were considered important contributors (Fig 6B). Compared with the summer grapes, the winter grapes displayed a distinct and significant upregulation of the unigenes (CCD, VIT_02s0087g00910; VIT_02s0087g00930), beta-carotene 3-hydroxylase (BCH, VIT_02s0025g00240), glutathione S-transferase (GST, VIT_15s0024g01540; VIT_12s0028g00920; VIT_12s0028g00930), flavonoid 3’5’-hydroxylase (F3’5’H, VIT_06s0009g03050; VIT_06s0009g02810), flavonol synthase (FLS, VIT_18s0001g03430), flavonoid 3-hydroxylase (F3H, VIT_18s0001g14310) and lycopene-beta-cyclase (LBCY, VIT_18s0001g14310). In contrast, the summer grapes presented upregulated levels of stilbene synthase (STS, VIT_16s0100g01100; VIT_16s0100g01200; VIT_16s0100g01150; VIT_16s0100g00750), caffeic acid 3-*O*-methyltransferase (COMT, VIT_03s0063g00140), phenylalanine ammonialyase (PAL, VIT_08s0040g01710), NCED (VIT_19s0093g00550) and dihydroflavonol reductase (DFR, VIT_16s0039g02350). The GO annotations of the transcripts were supplemented in the supplementary data (S2 Table).

### Transcriptomic changes in the carotenoid biosynthesis pathway under a two-crop-a-year cultivation system

**Fig 7:**
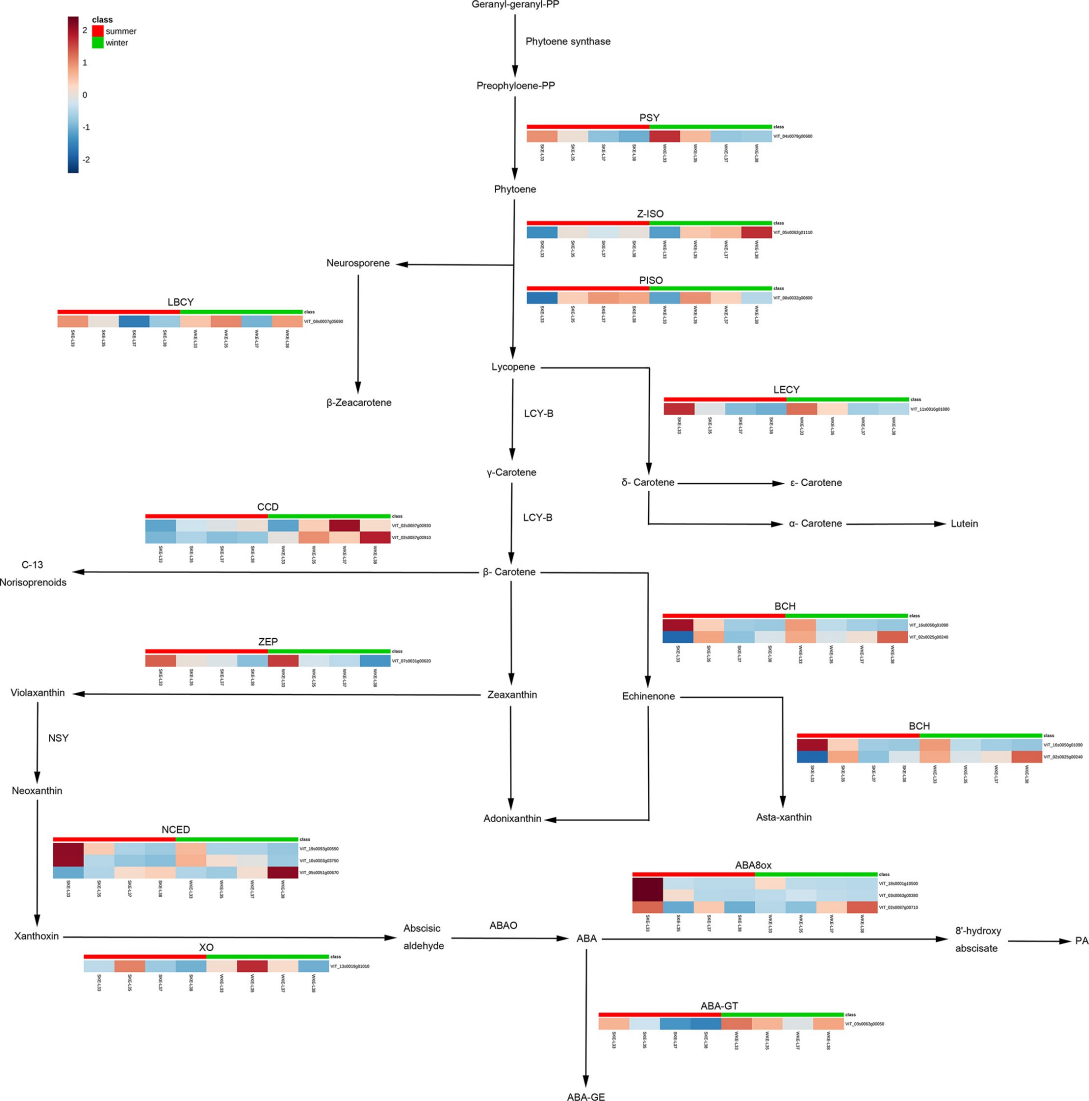
Transcriptomic profile of the structural genes involved in the carotenoid and ABA biosynthetic pathways in summer and winter grape berries. PSY, phytoene synthase; Z-ISO, zeta-carotene isomerase; PISO, prolycopene isomerase; LBCY, lycopene-beta-cyclase; LECY, lycopene epsilon-cyclase; CCD, carotenoid cleavage dioxygenase; ZEP, zeaxanthin epoxidase; NSY, neoxanthin synthase; NCED, 9-*cis*-epoxycarotenoid dioxygenase; BCH, beta-carotene 3-hydroxylase; XO, xanthosin dehydrogenase; ABA8ox, ABA 8’-hydroxylase; ABAO, abscisic-aldehyde oxidase; ABA-GE, ABA glucosyl-ester; ABA-GT, ABA glucosyltransferase; PA, phaseic acid. Each square in the heatmap located beside its gene names corresponds to the average FPKM value of the gene in each sample, as illustrated in the legend. SK, summer ‘Kyoho’; WK, winter ‘Kyoho’.

To explore the differences in gene expression patterns in the two seasons, a Kyoto Encyclopedia of Genes and Genomes (KEGG, http://www.genome.jp/kegg/) pathway enrichment analysis revealed that 18 unigenes encoding 12 enzymes were involved in carotenoid and ABA metabolism (Fig 7).

In this study, the expression of most of the unigenes participating in carotenoid biosynthesis peaked at E-L 31 or E-L 35, which are proposed to encompass the period of large-scale carotenoid production. However, the unigene encoding zeta-carotene isomerase (Z-ISO, EC:5.2.1.12) presented its maximum FPKM level at E-L 38. PSY (EC:2.5.1.32), Z-ISO, prolycopene isomerase (PISO, EC:5.2.1.13), LBCY (EC:5.5.1.19) and lycopene epsilon-cyclase (LECY, EC:5.5.1.18) were found to be involved in the upstream flux of carotenoid synthesis, and compared with that in the summer grapes, the expression of all the unigenes in the winter grapes was upregulated at the E-L 35, 37 and 38 stages. These results might suggest that the carotenoids contents in the winter grapes higher than summer grapes.

The downstream flux of carotenoid metabolism in grape berries includes the synthesis of norisoprenoids, a group of potent flavor and aromatic compounds. In plants, CCD (EC:1.13.11.51) catalyzes the breakdown of carotenoids into volatile norisoprenoids [7, 25]. Two CCD unigenes were identified and exhibited similar expression patterns between the two crop growing seasons, and compared with that in the summer grapes, the expression of all CCD members in the winter grapes was upregulated throughout the whole period.

In our study, one unigene annotated as *ZEP* and three annotated as *NCED* were identified. The ZEP unigenes showed similar expression profiles between the two crop growing seasons; compared with that at other stages, the expression of these unigenes at E-L 33 was upregulated. Young et al. [25] also reported a similar decreased expression pattern for two *ZEP*s from the green to harvest stages. In the present study, the three *NCED* transcripts exhibited different expression profiles. The *NCED6* expression level increased gradually in both the summer and winter grapes throughout their development, and compared with that in the summer grapes, *NCED6* expression in winter grapes was upregulated at E-L 38. Expression of the *NCED1* and *NCED2* transcripts peaked at E-L 33, and their expression levels in the summer grapes were higher than those in the winter grapes at E-L 33.

Compared with those in summer grapes, the expression levels of xanthosin dehydrogenase (XO, EC:1.1.1.288) and ABA glucosyltransferase (ABA-GT, EC:2.4.1.263) in winter grapes were upregulated throughout the whole period. Most transcripts of ABA 8’-hydroxylase (ABA8ox, EC:1.14.13.93) and BCH (EC:1.14.13.129) were expressed the most at E-L 33 for summer.

### Transcriptomic changes in the phenolic biosynthetic pathway under a two-crop-a-year cultivation system

**Fig 8:**
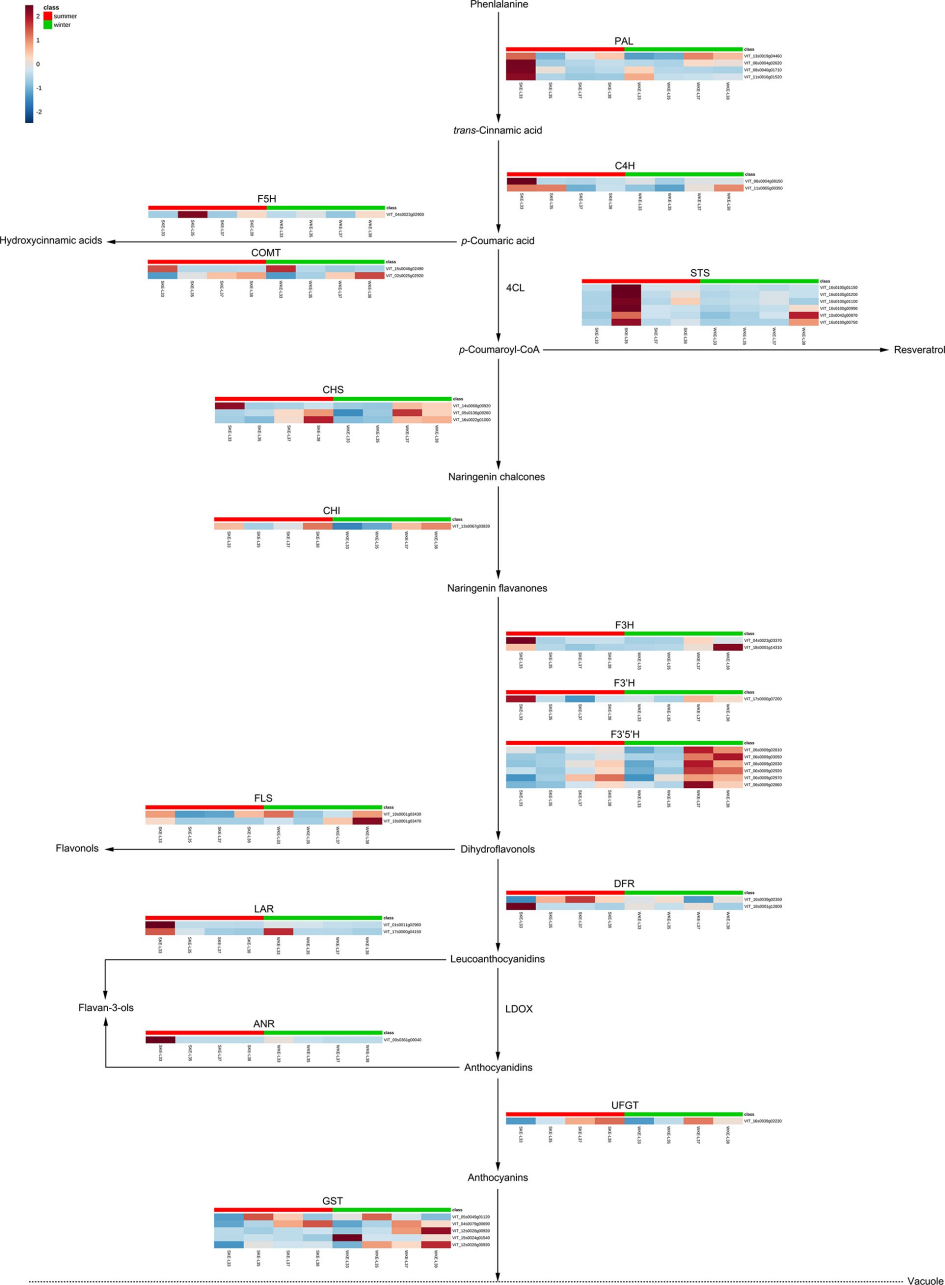
Transcriptomic profile of the structural genes involved in phenolic biosynthesis in summer and winter grape berries. PAL, phenylalanine ammonia-lyase; C4H, *trans*-cinnamate 4-monooxygenase; COMT, caffeic acid 3-*O*-methyltransferase; F5H, ferulate-5-hydroxylase; 4CL, 4-coumarate: CoA ligase; STS, stilbene synthase; CHS, chalcone synthase; CHI, chalcone isomerase; F3H, flavonoid 3-hydroxylase; F3’H, flavonoid 3’-hydroxylase; F3’5’H, flavonoid 3’,5’-hydroxylase; FLS, flavonol synthase; DFR, dihydroflavonol reductase; LAR, leucoanthocyanidin reductase; LDOX, leucoanthocyanidin dioxygenase; ANR, anthocyanidin reductase; UFGT, UDP-glucose: flavonoid 3-*O*-glucosyltransferase; GST, glutathione S-transferase. Each square in the heatmap located beside its gene names corresponds to the average FPKM value of the gene in each sample, as shown in the legend. SK, summer ‘Kyoho’; WK, winter ‘Kyoho’.

To investigate the differences in phenylpropanoid/flavonoid biosynthesis pathway-related structural genes between the summer and winter grapes at four distinct developmental stages (E-L 33, 35, 37, and 38) during the 2016 growing season, we used RNA-seq to characterize the changes in gene expression at the transcript level (Fig 8). The results showed that the expression of the general structural genes of the phenylpropanoid metabolic pathways, which included PAL (EC:4.3.1.24), trans-cinnamate 4-monooxygenase (C4H, EC:1.14.13.11), chalcone synthase (CHS, EC:2.3.1.74), F3H (EC:1.14.11.9), flavonoid 3’-hydroxylase (F3’H, EC: 2.3.1.133), DFR (EC:1.1.1.219), leucoanthocyanidin reductase 1 (LAR1, EC:1.17.1.3) and anthocyanidin reductase (ANR, EC:1.3.1.77), was significantly or moderately upregulated in the summer grapes at the E-L 33 stage.

STS is a pivotal enzyme that catalyzes the biosynthesis of resveratrol and is known to be induced by ultraviolet (UV) irradiation and *Botrytis cinerea* infection [26]. Compared with that in the winter grapes, the expression of almost all STS (EC 2.3.1.74) members in the summer grapes was significantly upregulated at the E-L 35 stage (the beginning of veraison).

The hydroxylation pattern of flavonoids is known to be mediated by the enzymatic activity of F3’H (EC 1.14.13.21), F3H (EC:1.14.11.9) and flavonoid 3’,5’-hydroxylase (F3’5’H, EC:1.14.13.88), which catalyze the hydroxylation of naringenin and dihydrokaempferol at the 3’, 3’ and 3’,5’ positions of the B-ring, respectively [27–28]. Our results showed that, compared with that in the summer grapes, the transcriptional abundance of several F3’5’H, F3’H and F3H family members in the winter grapes was significantly upregulated at the E-L 37 and E-L 38 stages. This result suggested that many more flavonoids containing flavonols, flavan-3-ols and anthocyanins would be synthesized in winter grapes than in summer grapes at the mature stage. Compared with those during the summer ripening of berries, the higher sunshine duration and lower rainfall during the winter ripening of berries were presumed to result in greater expression of some members of the F3’5’H, F3’H and F3H gene families in berries at E-L 37 and 38 stages and subsequently might contribute to the greater concentration of flavonoids.

Furthermore, FLS is responsible for catalyzing the formation of dihydroflavonols into flavonols. Two FLS unigenes exhibited different variation trends between developmental stages of the two crops, and higher expression levels were found in winter grapes than in summer grapes at each stage. LAR and ANR are key regulators of flavan-3-ol and proanthocyanidin biosynthesis [20], and the expression levels of both were highest at E-L 33 for both crops, after which the levels decreased sharply from veraison (E-L 35) to harvest (E-L 38). In addition, the expression of both *LAR* and *ANR* in the winter grapes was upregulated at E-L 33.

UDP-glucose: flavonoid 3-*O*-glucosyltransferase (UFGT) can catalyze the formation of anthocyanin-3-*O*-glucosides, and 5GT is critical for the synthesis of anthocyanin-3,5-*O*-diglucosides [29]. In our study, the expression of *UFGT* was higher at the later stages of grape maturity. Members of the GST family are believed to participate in vacuolar trafficking and the sequestration of anthocyanins from the endoplasmic reticulum to the vacuole [30]. In the present research, the expression of *GST* was significantly upregulated in winter grapes at the E-L 33, 35 and 38 stages.

## Discussion

The metabolism of carotenoids and phenols in grapes is influenced by variety, environmental factors, developmental stage, and plant hormone regulation. In recent years, transcriptomic studies involving secondary metabolites that respond to internal and external factors have been prevalent [20,31–34]. A two-crop-a-year cultivation system based on innovations in terms of pruning and pregermination was developed in southern China. Some works discussed the two-crop-a-year cultivation system in wine grape cultivars, such as 'NW196' (*V*. *quinquangularis* Rehd. × *V*. *vinifera* L.), ‘Cabernet Sauvignon’ (*V*. *vinifera* L.), ‘Riesling’ (*V*. *vinifera* L.) [12, 35]. ‘Kyoho’ was the first variety subjected to two-crop-a-year techniques in southern China. Here, the transcriptional expression patterns of phenols and carotenoids in ‘Kyoho’ grapes under a two-crop-a-year cultivation system were analyzed and compared for the first time. To our knowledge, this is the first study of the transcriptomic sequencing of two-crop-a-year ‘Kyoho’ grapes, providing a theoretical basis for future research on the quality of grapes grown during different growing seasons.

The climatic conditions of summer and winter grapes differed greatly. During the first growing season, the temperature gradually increased from germination until harvest. However, the second crop showed the opposite results. During berry development, the number of sunshine hours was clearly greater for the winter grapes than for the summer grapes. Additionally, the rainfall during the summer growing season was much greater than that during the winter growing season. Therefore, the differences in climatic factors strongly affect the variation in quality-related indicators between the two crops. During the maturation of grapes, increased sugar contents are favored by sufficient sunlight [36]. On the other hand, excessive humidity is unfavorable to sugar accumulation [37]. Thus, compared with the summer grapes, the winter grapes had a higher TSS content in the present study because of lower amounts of rainfall and richer sunlight conditions.

Carotenoids are precursors of C_13_-norisoprenoids and ABA, and when they occur mainly as glycoconjugated forms, C_13_-norisoprenoids contribute to the characteristic aromas of many varieties [38–39]. The carotenoids were mostly synthesized from E-L 33 until E-L 35, after which they degraded. The genes encoding nearly all the enzymes of carotenoid biosynthesis in plants have been identified [17]. Phytoene synthase (PSY), CCD, and NCED are widely believed to be the most important regulatory nodes in the separate biosyntheses of carotenoids, norisoprenoids, and ABA; these nodes are tightly controlled by environmental factors and development stage [23, 40]; compared with those in the summer grapes, these unigenes in the winter grapes were upregulated at grape-ripening stage. The expression of *PSY* is induced by light, water deficit, and hormones [40]. In the present study, compared with that in the summer grapes, the expression of *PSY* in the winter grapes was upregulated at the E-L 35, 37 and 38 stages as a result of drier climatic conditions and more sunlight during the winter season. In the present study, the transcriptional analysis of CCD genes in the two-crop-a-year grape berries revealed that, compared with that in the summer grapes, the expression of two unigenes in the winter grapes was upregulated. This observation was in agreement with the upregulated expression of *CCD* under much drier climatic conditions [17]. ABA is a plant hormone involved in environmental stress responses, and the production of ABA represents another flux of carotenoids within plants. The epoxidation of zeaxanthin to violaxanthin catalyzed by zeaxanthin epoxidase (ZEP) and the oxidative cleavage by NCED are two crucial steps in ABA biosynthesis, whereas ABA8ox and ABA-GT are the major enzymes for ABA glucosyl-ester (ABA-GE) and phaseic acid (PA) [17]. The expression patterns of *NCED* were similar between the summer and winter grapes, but the three transcripts showed different expression profiles during the different developmental stages. ABA8ox was predominantly expressed in the summer grapes at E-L 33, and ABA-GT expression peaked in the winter grapes at the same stage, indicating that ABA mainly broke down to PA in the summer grapes and was stored as ABA-GE in the winter grapes.

Longer sunshine hours and stronger light intensity result in relatively greater amounts of carotenoids synthesized at the green stage and lower amounts synthesized at the harvest stage [17]. Furthermore, many studies have shown that water deficit can increase carotenoid and norisoprenoid contents in grape berries [41]. In the present study, compared with those during first growing season, the green berry and veraison stages during the second growing season received lower amounts of rainfall but had a longer sunlight duration. These phenomena might explain the more active carotenoid and norisoprenoid synthesis pathway in the winter grapes during E-L 33 and E-L 35. Notably, these environmental factors are hard to separate. Thus, carotenoid metabolism is influenced by many integrated factors rather than by a single factor [17].

Phenolic compounds, mainly phenolic acids, stilbenes and flavonoids, are among the most abundant secondary metabolites in grapes. Phenolic acids accumulate in grape berry skin and flesh, while stilbenes have been detected in the skin, fruit flesh and seeds [20, 42]. Phenolic compounds are synthesized via the phenylpropanoid/flavonoid pathway. STS, or resveratrol synthase (EC 2.3.1.95), catalyzes the formation of resveratrol from *p*-coumaroyl-CoA; resveratrol is related to biotic and abiotic stresses in plants [43]. In the present study, compared with that in winter grapes, the expression of six STS unigenes in summer grapes was upregulated. Whether this difference was caused by sharp temperature increases at the veraison stage for summer grapes, needs to be further researched.

Flavan-3-ols, which share common upstream steps with both flavonols and anthocyanins, accumulated from E-L 27 (fruit set) until E-L 35 (veraison), after which they decreased [22]. In the present study, the expression of both *LAR* and *DFR* was highest at E-L 33 for both crops, and both genes were upregulated in the summer grapes. However, anthocyanins in winter ‘Kyoho’ grapes did not accumulate at the expense of flavan-3-ols in previous study [12], even though both compounds share common precursors. Thus, this finding may be associated with higher gene expression levels upstream of the phenolic biosynthesis pathway in winter grapes than in summer grapes.

F3’5’H, F3’H and F3H catalyze naringenin flavanones to form flavonoids containing flavonols, flavan-3-ols and anthocyanins [20]. The expression levels of *F3’5’H*, *F3’H* and *F3H* were upregulated in winter grapes at the E-L 37 and E-L 38 stages. These results also indicate that many more flavonoids accumulate in winter grapes than in summer grapes.

As a hybrid of *V*. *labrusca* and *V*. *vinifera*, ‘Kyoho’ grapes contain 3-*O*-glucosides and 3,5-*O*-diglucosides in their skin [11]. Although the transcripts of *UFGT* showed higher expression levels at some stages in the summer grapes than in the winter grapes, transcripts of *GST* were more abundant in the winter grapes than in the summer grapes at the E-L 33, 35 and 38 stages. Previous research has shown that, compared with summer grapes, winter grapes contain greater amounts of anthocyanins [12]. High temperature (35°C days) from veraison to harvest reduced the total anthocyanin content to less than half [44–45]. This conclusion can be explained by the higher number of extreme-temperature (> 35°C) days during the summer grape-ripening stage.

## Conclusions

The two-crop-a-year cultivation system is based on unique weather conditions in southern China. This work investigated the transcriptomic dynamics of ‘Kyoho’, which was the first variety cultivated via this technique. These results mainly revealed differences in the transcriptional expression patterns of phenols and carotenoids in ‘Kyoho’ grapes during the four stages of grape berry development in a two-crop-a-year cultivation system. The results showed that the second crop produced winter grapes that were smaller than the summer grapes and had greater contents of TSS and TA. Compared with the summer grapes, the winter grapes overall showed higher expression levels of genes required for the synthesis of carotenoids, which could be attributed to lower rainfall and much more abundant sunlight during the second growing season. In addition, the expression of some genes involved in the upstream portion of the phenylpropanoid/flavonoid pathway was upregulated in the summer grapes at E-L 33 and E-L 35. Although the expression of some genes directly related to the synthesis of flavan-3-ols and anthocyanidins was upregulated in the summer grapes at E-L 37 and E-L 38, the expression of three hydroxylase genes and *GST* was higher in the winter grapes than in the summer grapes. All of these factors could result in the production of less flavonoids and could be explained by extremely hot weather during the mature stage of the first crop. We anticipate that the present study will enrich theoretical research on two-crop-a-year cultivation systems and will facilitate additional investigations into the differences in grape berry quality between two crops grown during the same year.

## Supporting information

S1 Table List of the primers used for quantitative real-time RT-PCR validation experiments. (PDF)

S2 Table The GO annotations of the transcripts were supplemented for Fig 6B. (PDF)
