# Supplementary material for: Comparison of transcriptional expression patterns of phenols and carotenoids in ‘Kyoho’ grapes under a two-crop-a-year cultivation system
Source: PLoS One. 2019 Jan 10;14(1):e0210322. doi: 10.1371/journal.pone.0210322 (PMC6328245; doi:10.1371/journal.pone.0210322)
Supplement: S1 Table — (PDF) [file pone.0210322.s001.pdf]

Gene name	Forward primer(5’-3’)	Reverse primer(5’-3’)
PSY1	TGGGCAATTTATGTGTGGTGCAG	GCCTTGGGTGACATGTGTGAAG
PSY2	TCGATGTGGTGAAGTTTGTGCAG	TCAGGTGTCATCAGCATTGTTCCC
CCD4a	TGAAGCTGGACGTGTCCCAAAC	TAGCACCCTGGTCCATACATCCTG
CCD4b	GTTGGAATCATCCCTCGTT	CCTCGTCCCAGGCGTTA
NCED	GCAGAGGACGAGAGTGTAAAGGA	GCAGAGTAAAAACACATGAAGCTAGTG
LBCY	ATGGATGCTGCAATTGGCCTTC	TCCCGACTGGGTCATGGATTAAG
ANR	CTTGATGGGACAGGTCTGGT	TGTCTTGGAGGCAGGATAGC
F3’H	ACTCATGGTGCACCCACG	CACAAAACTACTAAACTCGCTC
F3’5’H	AAACCGCTCAGACCAAAACC	ACTAAGCCACAGGAAACTAA
STS1	CATCGGTGAAGGATTGGATTG	ACATTGAAGGGTCGAGCATTC
LAR1	CTCCAACGGATTTCTTCC	CGTCCACTGTTTTCATCGT
LAR2	TAAACGAGCTGGCATCAC	GCAGCGGCTAGTAGGTCA
DFR	GAAACCTGTAGATGGCAAGA	GGCCAAATCAAACTACCAGA
UFGT	GGGATGGTAATGGCTGTGG	ACATGGGTGGAGAGTGAGTT
Ubiquitin	GTGGTATTATTGAGCCATCCTT	AACCTCCAATCCAGTCATCTAC
Actin	GCATCCCTCAGCACCTTCCAGCAG	CCACCTCAACACATCTCCATGTCAA
