# Supplementary material for: Comparison of transcriptional expression patterns of phenols and carotenoids in ‘Kyoho’ grapes under a two-crop-a-year cultivation system
Source: PLoS One. 2019 Jan 10;14(1):e0210322. doi: 10.1371/journal.pone.0210322 (PMC6328245; doi:10.1371/journal.pone.0210322)
Supplement: S2 Table — (PDF) [file pone.0210322.s002.pdf]

Gene ID	GO annotation
VIT_02s0087g00910	9-*cis*-epoxycarotenoid dioxygenase activity (GO:0045549)
VIT_02s0025g00240	Carotene metabolic process (GO:0016119)
VIT_15s0024g01540	Glutathione transferase activity(GO:0004364)
VIT_12s0028g00920	Glutathione transferase activity(GO:0004364)
VIT_12s0028g00930	Glutathione transferase activity(GO:0004364)
VIT_06s0009g03050	Flavonoid 3',5'-hydroxylase activity (GO:0033772)
VIT_06s0009g02810	Flavonoid 3',5'-hydroxylase activity (GO:0033772)
VIT_18s0001g03430	Flavonol synthase activity (GO:0045431)
VIT_16s0100g01100	Defense response (GO:0006952)
VIT_16s0100g01200	Defense response (GO:0006952)
VIT_03s0063g00140	Caffeic acid *O*-methyltransferase activity (GO:0042409)
VIT_05s0062g00640	Anthocyanidin 5-*O*-glucosyltransferase activity (GO:0080018)
VIT_16s0100g01150	Defense response (GO:0006952)
VIT_02s0087g00930	9-*cis*-epoxycarotenoid dioxygenase activity (GO:0045549)
VIT_08s0007g05690	Lycopene beta-cyclase activity (GO:0045436)
VIT_08s0040g01710	Phenylalanine ammonia-lyase activity (GO:0045548)
VIT_18s0001g14310	Flavonoid biosynthetic process (GO:009813)
VIT_19s0093g00550	9-*cis*-epoxycarotenoid dioxygenase activity (GO:0045549)
VIT_16s0100g00750	Defense response (GO:0006952)
VIT_16s0039g02350	Flavonoid biosynthetic process (GO:009813)
